# Easy-hard phase transition in parameter estimation for optical waveguides

**DOI:** 10.1038/s41598-020-74366-5

**Published:** 2020-10-15

**Authors:** Gunnar Claussen, Alexander K. Hartmann

**Affiliations:** Institut für Physik, Carl von Ossietzky Universität Oldenburg, 26129 Oldenburg, Germany

**Keywords:** Micro-optics, Characterization and analytical techniques, Phase transitions and critical phenomena

## Abstract

The determination of the parameters of cylindrical optical waveguides, e.g. the diameters $\vec {d}=(d_1,\ldots ,d_r)$ of *r* layers of (semi-) transparent optical fibres, can be executed by inverse evaluation of the scattering intensities that emerge under monochromatic illumination. The inverse problem can be solved by optimising the mismatch $R(\vec {d})$ between the measured and simulated scattering patterns. The global optimum corresponds to the correct parameter values. The mismatch $R(\vec {d})$ can be seen as an *energy landscape* as a function of the diameters. In this work, we study the structure of the energy landscape for different values of the complex refractive indices $\vec {n}$, for $r=1$ and $r=2$ layers. We find that for both values of *r*, depending on the values of $\vec {n}$, two very different types of energy landscapes exist, respectively. One type is dominated by one global minimum and the other type exhibits a multitude of local minima. From an algorithmic viewpoint, this corresponds to *easy* and *hard* phases, respectively. Our results indicate that the two phases are separated by sharp phase-transition lines and that the shape of these lines can be described by one “critical” exponent *b*, which depends slightly on *r*. Interestingly, the same exponent also describes the dependence of the number of local minima on the diameters. Thus, our findings are comparable to previous theoretical studies on easy-hard transitions in basic combinatorial optimisation or decision problems like *Travelling Salesperson* and *Satisfiability*. To our knowledge our results are the first indicating the existence of easy-hard transitions for a real-world optimisation problem of technological relevance.

## Introduction

Optical waveguides are of high technological relevance in many fields like fast communication, transfer of energy or illumination in medical and biological applications^1^ and therefore still much under investigation, in fundamental and applied studies. One important task during production is to control, in particular measure, the diameter of the waveguides. This allows, e.g., to minimise the loss when connecting two waveguides. In the past, several methods have been used, which are based on measuring specific properties of diffraction patterns^2–5^. Other approaches take more properties into account, but suffer from special assumptions or computational problems^6–8^. Therefore, recently, a method has been introduced^9^ which takes the full diffraction pattern into account and overcomes these problems.

To be more precise, in this recent work an algorithm was devised which is intended to determine the layer diameters $\vec {d} =(d_1,d_2,\ldots ,d_r)$ of a circular stratified cylinder consisting of *r* concentric layers of transparent materials with the respective refractive indices $n_s$ ($s=1,\ldots ,r$). These cylinders were to be illuminated by monochromatic light of wavelength $\lambda$ under perpendicular incidence in an outer medium of refractive index $n_m \approx 1$, and the lateral intensity far-field pattern $u_\infty$ was measured on a line of CCD detectors placed at a distance of $y_{\text {CCD}}$ from the cylinder centre.

The basic idea of this approach is typical for an *inverse problem*^10^. Usually, inverse problems are treated by numerically *forward* calculating some output for a model of the given system. Often the output is an observed scattering, absorption or interference pattern. The computed output is compared to the experimentally measured one. By repeatedly adjusting the model parameters and recalculating the forward problem until the calculated pattern resembles the observed one, the inverse problem is solved. Hence, inverse problems are typically very hard problems. In many different application fields, a lot of effort has been put in to solve them. For the present problem this means we want to determine the diameters $\vec {d}$ such that a numerically computed inference pattern is as similar as possible to the measured pattern $u_\infty =u_\infty (\vec {x})$, where $\vec {x}$ are spatial positions where the pattern is evaluated. Here we use a finite number *N* of positions where the scattering pattern is measured hence $u_\infty$ is a vector of size *N*. The algorithm starts at iteration step $n=0$ with initial values of the parameter set $\vec {d}^{(0)}$. One computes numerically the scattering pattern $F(\vec {x}|\vec {d}^{(0)})$ for this set. For technical reasons, also the derivative $F'(\vec {x}|\vec {d}^{(0)})$ will be needed. In each step *n*, the adjustment of the parameters $\vec {d}^{(n)}$ is done as to minimise the norm $R(\vec {d}) := ||F(\vec {x}|\vec {d}) - u_\infty ||_2$. Here, we used the *iteratively regularised Gauss-Newton* (IRGN) algorithm, which calculates each time from $F(\vec {x}|\vec {d}^{(n)})$ and $F'(\vec {x}|\vec {d}^{(n)})$ the step alteration $h_n$, so that $\vec {d}^{(n+1)} = \vec {d}^{(n)} + h_n$ iteratively^11^. As a condition for the termination of the algorithm, a certain value $R_0$ of the norm *R* was given as a threshold. Thus, to understand the nature of this optimisation problem, it appeared useful to have insight into the topography of the *energy landscape* on which the algorithm operates. In this work we will study the energy landscape not with respect to actually measured patterns, but with respect to numerically computed patterns $F(\vec {x}|\vec {d}_{\text {real}})$ for a set $\vec {d}_{\text {real}}$ of known parameters. Still, for easier discrimination, we call this the *measured* pattern. In a previous work^9^ it could be pointed out easily that for transparent cylinders (${\text {Im}}(n_s) \equiv 0$) this landscape exhibits local minima, which let the IRGN algorithm terminate wrongly. It could be observed that these minima are usually separated systematically by a distance in *d*-space that only depends on the relative refractive index $m_s = \frac{n_s}{n_{s+1}}$ or $m_s =\frac{n_s}{n_m}$ for the outermost layer, and thus with this knowledge it is feasible to utilise this property in order to allow the IRGN algorithm to traverse these local minima and terminate at the global minimum^9^. Concurrently, the Dividing Rectangles (DiRect) algorithm was also considered, and with this being an algorithm dedicated to global optimisation, i.e., minimising an objective function from the onset^12^, it appeared that the treatment of our task as an optimisation problem of $R(\cdot )$ as an objective function is more than suitable.

However, one thing appeared striking right from the beginning: When considering a solid weak-transparent cylinder, the energy landscape given by *R*(*d*) over a range of diameters *d* exposes no local minima, but only one global minimum. From the computational viewpoint, this means the optimisation problem is “easy” to solve. Weak-transparent cylinder material is characterised by a high absorption constant $\kappa = {\text {Im}}(n_s)$, i.e., the presence of a nonzero imaginary part in the refractive index $n_s$. For the case of full transparency, the energy landscape turned out to be more complex, exhibiting several local minima. Thus, finding the global minimum is “hard”. What happens in between the full transparent and weak-transparent states was not known, but it appeared possible that there exists a sharp threshold in parameter space which separates the easy from the hard region, resembling a phase transition in physics. Such phase transitions with respect to computational complexity are mostly known^13–15^ for classical combinatorial optimisation problems which are studied in theoretical computational complexity, such as the *Vertex Cover* problem on random graphs^16–18^, the random *K-Satisfiability* problem^19–22^, the *Number Partitioning* problem^23,24^, the *Travelling Salesperson* problem^25,26^, or the *Colouring* problem^27^. But such models typically are very abstract and based on discrete topologies (e.g., graphs, lattices, cells) only. To our knowledge there is no study of a real-world optimisation problem with respect to its computational hardness and a related phase transition. It is the aim of the present work to fill this gap: our model of diffraction patterns in waveguides in turn is closely linked to the analytical description of real-world phenomena with a continuous support, i.e., the Lorenz–Mie theory for describing scattering of light on cylinders. Hence, observing a phase transition here unites theoretical and experimental physics in a certain way. Note also that here “hard” means that the problem will require typically an algorithmic running time which increases exponentially in the number of variables or degrees of freedom, which is the number *r* of layers here. Still, even for a fixed number of layers, it will turn out here much harder to find the global optimum in the hard phase compared to the easy phase.

Before moving over to our results, it should be remarked that treatments of inverse parameter estimation as optimisation problems are actually scarce. The closest match so far is the calculation of an estimator based on the differences in the number and angular positions of local extrema within two scattering patterns^7,8^. This, however, involves the computationally intricate task of calculating a look-up table in which necessary data for the calculation of these estimators are stored. It is also of no help that most studies in this field do not consider circular objects (such as infinite cylinders), but address the seemingly more complex tasks of shape reconstruction^28,29^ or positioning of a multitude of scatterers^30^—which in turn appears to have been a motivation to favour genetic or evolutionary algorithms for optimisation in such cases^31,32^. It should also be added also that research on this field hardly uses light from the visual range of the electromagnetic spectrum, but microwave imaging appears to be favoured instead^33^, if not even acoustic tomography is used^29^. Hence, it appears that our study offers a completely novel approach.

Also we want to stress that our study is rather a basic-research one. We are interested in the properties and energy-landscape structure of the diameter-determination optimisation problem from a fundamental point of view. The diameter determination, as it is actually performed in labs, can be seen as an experimental realisation of rather theoretical optimisation problems like *Vertex Cover* or *Maximum Satisfiability*. Thus, we do not claim that our results will (immediately) lead to better algorithms or to better optical waveguides. Our work is motivated by experimental findings, but not aimed to improve experiments. This relation between theory and practice is probably like that a better theoretical understanding of the ferromagnet-paramagnet transition and the calculation of its characteristic critical exponents do not (immediately) lead to magnets with better properties.

This paper is organised as follows. The first section will introduce the theoretical background and the used methods. This encompasses the notion how specification of the norm $R(\cdot ) = ||F(\vec {x}|\vec {d}^{(n)}) - u_\infty ||$ as the objective function of an optimisation algorithm can be interpreted as an energy landscape. Further details regarding the calculation of scattering patterns and usage of the IRGN algorithm are given in supplementary information. The following section will present the results for homogeneous fibres, i.e., the one-dimensional optimisation problem. This includes the critical behaviour of the norm $||F(\vec {x}|\vec {d}^{(n)}) - u_\infty ||$ as a function of diameter *d* under increase of the absorption coefficient $\kappa$ itself as well as the results of the IRGN algorithm for this case. Ultimately, the fourth section will also show how this critical behaviour affects the two-dimensional energy landscapes given by $R(\vec {d})$ for cylinders of two layers. As will be seen, the critical behaviour can reduce the dimension of this optimisation problem from two to one, indicating a phase transition regarding the complexity.

## Theory and methods

### Diameter estimation from scattering intensity patterns as an optimisation problem

**Figure 1:**
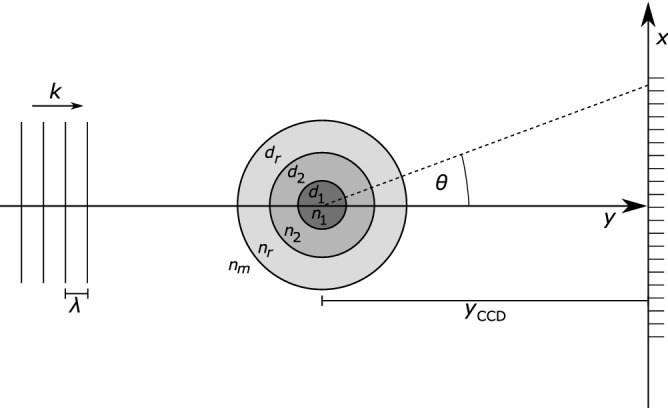
General setup for the generation of scattering patterns. Monochromatic light of wavelength $\lambda$ illuminates the scattering cylinder (*r* layers of diameter $\vec {d} = (d_1,\ldots ,d_r)$ and refractive index $n_s$, respectively) perpendicularly. The scattered light is then detected at a number of coordinates $\vec {x}$ representing CCD cells arranged in a line.

This section is intended to demonstrate how the estimation of layer diameters $\vec {d}$ of a circular cylinder from evaluation of the intensity patterns that can be observed under perpendicular illumination can be understood as an optimisation problem consisting in finding the minimum of an objective function $R(\vec {d})$. The geometric setup of this case is illustrated in Fig. 1. Illumination of the cylinder with monochromatic light of wavelength $\lambda$ causes diffraction and refraction on the boundaries of the cylinder layers. Interference scattering patterns $F(\vec {x}|\vec {d})$ emerge that can be measured as intensities on a line array of CCD cells, denoted by their positions $\vec {x}$. The diameters $\vec {d}$ constitute the set of parameters of the scattering setup that are yet to be estimated. The actual scattering intensities can be calculated according to Lorenz–Mie theory^34^, and this scheme is not only the topic of multiple textbooks on optics^35,36^, but also has been used and outlined in an earlier article on algorithms actually dedicated to parameter estimation^9^. Thus, instead of recounting these expressions yet again, we instead leave it with a reference to these sources and the supplementary material, where the respective equations are given nevertheless.

For parameter determination, we use the iteratively regularised Gauss-Newton algorithm, which iteratively calculates the best matching parameters for a given scattering intensity pattern $u_\infty$^9,11^. Since this algorithm has also been researched exhaustively in the past, we again refer to the original sources and the supplementary material for a formal introduction of this algorithm. With respect to the topic of this article, we however would like to stress several of its properties: Consider that the algorithm already takes into account the norm $||F(\vec {x}|\vec {d}^{(n)})-u_\infty ||_2$ between the calculated intensity $F(\vec {x}|\vec {d}^{(n)})$ and the input pattern $u_\infty$ for its termination condition: If this quantity drops below a given threshold, the algorithm terminates. Thus, the algorithm is highly efficient in lowering this quantity, but also prone to wrongful termination in local minima. But as we explicitly seek for local minima here, we are not required to apply any of the modifications proposed earlier^9^ and instead may use the algorithm in its basic form.

This maybe already gives a hint on the general methodology used in this article. In the following we will study energy landscapes $R(\vec {d}) = ||F(\vec {x}|\vec {d})-F(\vec {x}|\vec {d}_{\text {real}})||$ over the considered diameters $q=\vec {d}$ with respect to a given set of “real” or reference diameters $\vec {d}_{\text {real}}$. For just evaluating the energy landscapes, we only have to evaluate $R(\vec {d})$ for a grid of many values of $\vec {d}$. When actually trying to find the “best” parameters in a practical application with, e.g., the IRGN algorithm, one seeks to minimise such a norm, which hence could also be used as the objective function for the DiRect algorithm^9^. Therefore, we need a meaningful definition of the norm $||\cdot ||$. Colloquially, this corresponds to choosing a (scalar) measure to describe the similarity between the two scattering patterns $F(\vec {x}|\vec {d})$ and $F(\vec {x}|\vec {d}_{\text {real}})$. Note also that such a norm by definition is always greater than or equal to zero, and the zero point corresponds exactly to $\vec {d} \equiv \vec {d}_{\text {real}}$.

We might think of different definitions of this measure, such as the maximum norm $||F(\vec {x}|\vec {d})-F(\vec {x}|\vec {d}_{\text {real}})||_{\max } = \max _{\vec {x}} |F(\vec {x}|\vec {d})-F(\vec {x}|\vec {d}_{\text {real}})|$, where the maximisation is performed over all measured positions of the intensity pattern. Alternatively one could take the Manhattan norm $||F(\vec {x}|\vec {d})-F(\vec {x}|\vec {d}_{\text {real}})||_1 = \sum _{\vec {x}} |F(\vec {x}|\vec {d})-F(\vec {x}|\vec {d}_{\text {real}})|$, but ultimately we stick to the Euclidean norm

1 $$\begin{aligned} ||F(\vec {x}|\vec {d})-F(\vec {x}|\vec {d}_{\text {real}})||_2 = \sqrt{ \sum _{\vec {x}} \left( F(\vec {x}|\vec {d})-F(\vec {x}|\vec {d}_{\text {real}})\right) ^2}. \end{aligned}$$

The main reasons for this choice are two mutually dependent properties: First, the Euclidean norm is tolerant towards differences. These are evened out through the summation of squares, instead of dominating other values (as in the maximum norm). Therefore, the course of $R(\vec {d})$ is smooth and continuous (note that the intensity patterns themselves are generally also continuous and bounded). Second, this appears to hold especially in the presence of noise: A noisy deviation from the “actual” course of the scattering pattern is also evened out, while these deviations are merely carried over by the other norms.

Note that Onofri et al.^8^ proposed an estimator which neglects the intensity values completely and only compares the angular positions of extrema within the scattering patterns. That estimator however is then constructed by multiplications of the corresponding absolute angular differences and also includes a “discrimination factor” in case the numbers of extrema differ. Usage of this estimator would lead to a highly non-continuous, if not discrete, energy landscape that spans over several magnitudes and thus is highly unsuitable as an objective function for an optimisation problem.

### Periodic spacing of local minima in the objective function

The application of the IRGN algorithm or any “local-descent” optimisation algorithm to scattering on transparent cylinders requires the usage of repeated restarts with random initial conditions, as the norm $||F(\vec {x}|\vec {d}^{(n)})-u_\infty ||_2$, which is equivalent to the objective function $R(\cdot )$ of the optimisation problem, exposes a number of local minima, where the algorithm may terminate wrongly. However, for the one-layer case it has been found that these minima of the landscape $R(d_1)$ are separated by a fixed periodicity, and thus it is possible to exploit this property in order to allow for a systematic traversal of these local minima^9^. As we will see below, this holds to some extent for $r > 1$ layers as well, with the minima then being represented by a series of “tilted trenches” in the energy landscape represented by $R(\cdot )$. Actually, also for the case for $r>1$ without absorption, one can calculate^37^ the distance of the minima within any one-dimensional slice of the energy landscape, i.e., when varying only one diameter value. Nevertheless, this distance only depends on the wavelength and on the difference of the refractive indices of neighbouring layers. Also, the energy landscape is not simply a product of the periodic patterns in the different dimensions. Thus this does not help a lot in actually determining the global minimum.

In particular in presence of absorption and due to the dependence on several diameter values, the energy landscapes in particular for $r>1$ layers will exhibit a complex behaviour, as we will see below. Nevertheless, we start in the following section with the case of one layer, i.e., an one-dimensional energy landscape.

## Results for the homogeneous (1-dimensional) fibre

Now, we want to show how the addition of absorption affects the complexity of the optimisation problem which the IRGN or similar algorithms are tasked to solve (or not), beginning with homogeneous cylinders consisting of one single material only. Hence, the parameter set consists of the diameter only, $q=(d)$. To do so, we generally approach this property by two means:

1. On one hand, we sample a range $d \in [d_{\min }, d_{\max }], d_{\min }< d_{\text {real}} < d_{\max }$ with respect to a given reference diameter $d_{\text {real}}$, which in turn determines the reference pattern $F(\vec {x}|\vec {d}_{\text {real}})$. This we do for several values of the absorption coefficient $\kappa$. Each time we calculate the norm *R*(*d*) for each value *d* of the diameter in order to draw the energy landscape, and simply enumerate the local minima found within it. Besides the statistical information about the number of minima, we may also observe how (and where) the local minima disappear while the cylinder becomes more and more absorbing.
2. The other method involves multiple applications of the IRGN algorithm with different randomly drawn initial values $d_0 \in [d_{\min }, d_{\max }]$ also for each value of $\kappa$. In this case, the question is whether the result $\vec {d}_{\text {final}}$ of the IRGN algorithm meets the correct diameter $d_{\text {real}}$ with sufficient precision as required in waveguide manufacturing, i.e., $|d_{\text {final}} - d_{\text {real}}| < 0.1\, \upmu \hbox {m}$. Algorithm executions with a result of such precision are counted as “successful”, and the fraction of successful executions (of all considered random initial values) we call the *success ratio* $r_{\text {succ}}$. With local minima disappearing with increasing $\kappa$, we can expect the success ratio to climb as the zone of attraction of the global minimum grows successively larger.

Note that in the latter method the success ratio will never actually drop to zero as long as the range from which the initial value $d_0$ is drawn also covers the real diameter $d_{\text {real}}$. This would only be the case if this range deliberately excluded $d_{\text {real}}$ and the whole region between the adjacent local maxima. In turn, the success ratio might be deduced from the energy landscape (calculated in the first method) by measuring the *basin of attraction*, i.e., figuring out these two adjacent maxima and measuring the *d*-distance in between them.

### Setup and fundamental observations

Regarding the parameters of the setup, both approaches use the same parameters. Thus, we assume light of a wavelength of $\lambda = 636.7\,\text {nm}$ (typical of a Nd:YAG laser) in an outer medium with a refractive index of $n_m = 1$ (vacuum). As mentioned before, the scattering cylinder is circular and homogeneous, specified by its diameter $d_{\text {real}}$ and refractive index $n_s$, and placed at a distance of $y_{\text {CCD}} = 0.1\,\text {m}$ from the observing plane. Here, the scattering light is calculated at 200 points $\vec {x}$ along a line perpendicular to the optical axis, starting at $x = 0$ and separated by a spacing of $\delta x = 10\, \upmu \hbox {m}$, thus representing the part of a typical CCD array with sufficiently large intensities. Note that we only calculate one side of the scattering patterns, as Lorenz–Mie theory for this setup generally results in symmetrical scattering patterns for detecting lines perpendicular to the optical axis, and extending the pattern to the other side as well would affect the course of $R(\vec {d}) = ||F(\vec {x}|\vec {d})-F(\vec {x}|\vec {d}_{\text {real}})||_2$ by a multiplicative factor only, but not by the actual shape.

**Figure 2:**
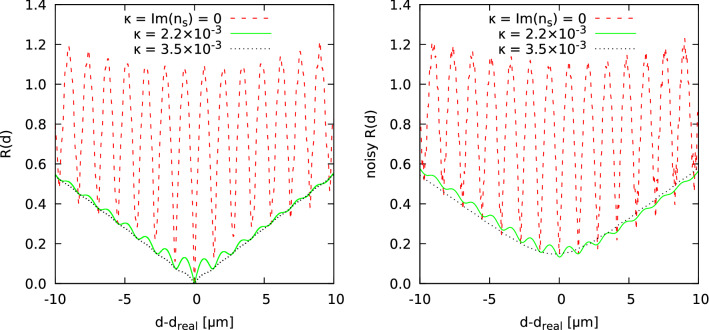
Examples for the behaviour of the energy landscape given by *R*(*d*) at $d_{\text {real}} = 100\, \upmu \hbox {m}$ under variation of the absorption coefficient $\kappa$. Left: pure measurement signal, right: noisy measurement signal. As can be seen, the transparent phase, i.e. $\kappa = 0$, is characterised by periodically spaced local minima, while the weak-transparent phase with $\kappa = 3.5\times 10^{-3}$ only exposes one single, global minimum. The local minima disappear one after another in the intermediate regime around $\kappa = 2.2\times 10^{-3}$. The case with noise leads to a shift of the optima up, and for $\kappa =3.5\times 10^{-3}$ the global optimum shifts also left away from the true radius value.

The real part of the refractive index $n_s$ we keep fixed at ${\text {Re}}(n_s) = 1.458$, the approximate value for fused silica ($\hbox {SiO}_2$) at the given wavelength $\lambda$ according to the Sellmeier equation. In turn, the imaginary part ${\text {Im}}(n_s) = \kappa$, is altered over different ranges to study the influence of absorption. As we will see below, the actual ranges where the behaviour changes depend on the given cylinder diameter $d_{\text {real}}$.

For explicit sampling of the energy landscapes, we sampled trial values for the diameter in a range of $d \in [d_{\text {real}} - 50\, \upmu \hbox {m}, d_{\text {real}} + 50\, \upmu \hbox {m}]$ with an interval of $\delta d = 0.1\, \upmu \hbox {m}$, which is sufficient to detect the presence of local minima, as the periodic distances between them are expected to be around $\delta d_{\min } \approx 2.18\lambda$^9^. The exact positions of the local minima can not be detected with rather coarse spacing used here, but we approximate these by application of cubic splines to the coarse sampled points, which have been found to be in good agreement with actual measurements that were sampled much finer ($\delta d = 1\,\text {nm}$). However, this is of not as much relevance here, as the only interesting property is the actual number of local minima observed over the range of diameters *d*.

Note also that for small reference diameters $d_{\text {real}}$, we excluded trial diameter values for $d < 30\, \upmu \hbox {m}$, as interference effects within the cylinders affect the scattering patterns for such small values and lead to odd behaviour of the norm. Larger reference diameters in excess of $d_{\text {real}} > 80\, \upmu \hbox {m}$ were obviously not affected by this.

The general property we put under detailed scrutiny is exemplified in Fig. 2: For low values of $\kappa$, the energy landscape is characterised by multiple local minima that are equally spaced along the *d*-axis^9^. Increasing $\kappa$ however alters this. The norm values *R* at local minima decrease slightly, those at local maxima decrease strongly, thus producing a mechanism which lets the corresponding local extrema disappear. Ultimately, this leads to an energy landscape typical for weak-transparent cylinders, which only features one global minimum.

A large number of local minima might lead to wrong local termination for local-optimisation algorithms such as the aforementioned IRGN algorithm. As for this, the complexity of the optimisation problem constituted in detecting the minimum of the landscape *R*(*d*) is relaxed with increasing value of $\kappa$: The landscape shown in Fig. 2 offers for a large enough value of $\kappa$ a global minimum only with a wide catchment range. As we will see next the disappearance of all non-global minima happens once ${\text {Im}}(n_s) = \kappa$ as the control parameter has crossed a certain threshold. This is the transition we want to study in more detail.

Note also that Fig. 2 gives a hint at another peculiar property of this transition. It can be seen that the local minima on the “right” side of the energy landscape, i.e., those for $d > d_{\text {real}}$, disappear first, while others on the “left” side, i.e for $d < d_{\text {real}}$ still persist. Hence, the transition might be treated separately for the both “sides” of the diameter range, although the energy landscape as a whole is thus dominated by the behaviour of the left side where the minima remain for higher values of $\kappa$. Note also that the receding of the local extrema appears to affect a “medium” region in the first place, while the local minima near $d_{\text {real}}$ as well as those for large distances $|d-d_{\text {real}}|$ persist much longer. In particular, it should be remarked that actually the two “neighbouring” local minima near $d_{\text {real}}$ disappear only lately, thus the aforementioned global solvability of the optimisation problem is primarily prohibited by the sheer presence of these two minima.

In real measurements, the signal will always we noisy. For this reason, we have also studied the case where the measurement signal was disturbed with a noise strength of 0.01, i.e., at any coordinate $\vec {x}$ a value from a Gaussian distribution with zero mean and a variance of 0.01 was added to the calculated scattering intensity $F(\vec {x}|\vec {d}_\text {real})$ of the input pattern. On the right of Fig. 2, we show the corresponding results. One first observes that the global optima and near local minima are shifted up. This is natural, because the presence of the noise leads immediately to differences between simulated and measured diffraction patterns even if the true radius is used for the simulated pattern. Furthermore, for the transparent and almost transparent cases, the optimisation algorithm can still find in principle the true radius, because the global minimum is still located at $d=d_{\text {real}}$. But for the weak transparent case, the global minimum is shifted to a somehow smaller radius. Thus, in this case no optimisation algorithm would be able to detect the true radius precisely.

### Sampling of local minima

For the first of the two approaches mentioned at the beginning of this section, we simply look at how the numbers of local minima develop under variation of $\kappa$ for different diameters $d_{\text {real}}$. The transition we seek for is marked by a more or less sharp, rapid decay in the number $N_{\min }$ of local minima which marks the change from “transparent” material and a more difficult optimisation problem to less transparent material and a simple optimisation problem such that even a local algorithm might solve globally in one run. Note however that the actual number of local extrema is also an extensive quantity which also depends on the actually considered range of trial values *d*. Nevertheless, having practical applications in mind, one would probably not use starting values $d_0$ for the IRGN algorithm which are very far from the actual diameter, so studying the number of observed minima $N_{\min }$ within a rather large range is sufficient to obtain an understanding of the energy landscapes.

**Figure 3:**
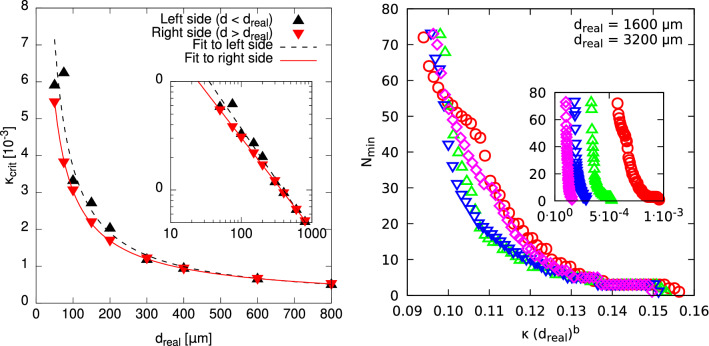
(left) Dependence of the absorption coefficient $\kappa _{\text {crit}}$ which marks the complete vanishing of local minima in the energy landscape as a function of the actual cylinder diameter $d_{\text {real}}$. It can be seen that the values behave according to the power-law relation given in Eq. (2), and once again both “sides” of the energy landscape behave slightly different. (inset) the same on a double-logarithmic scale. (right, inset) The total number of local minima $N_{\min }$ as a function of the absorption coefficient $\kappa$. (right, main plot) $N_{\min }$ as a function of the rescaled absorption coefficient $\kappa d_{\text {real}}^d$.

We define as the transition point the imaginary part value $\kappa _{\text {crit}}$ were upon increasing the absorption the first time the landscape exhibits no additional local minima. To be precise, $\kappa _{\text {crit}}$ is defined as the lowest value of absorption for which $N_{\min } = 1$ minima are observed. With this, the recorded values of $\kappa _{\text {crit}}$ obviously depend on the sampling resolution of $\kappa$. We have chosen a rather narrow sampling, as displayed in right of Fig. 3. The dependence $\kappa _{\text {crit}}(d_{\text {real}})$ is shown in the left of Fig. 3. Note that the disappearance of the local minima left and right of the global minimum are treated separately, but do not result in a great difference of $\kappa _{\text {crit}}$. The general behaviour of $\kappa _{\text {crit}}(d_{\text {real}})$ is reasonable: With increasing diameter, the overall absorption is increased, hence the critical value necessary to make the cylinder absorbing enough to observe only one minimum is reduced.

Note that Fig. 3 constitutes a kind of phase diagram: For cases where the parameter tuples $(d_{\text {real}},\kappa )$ lie below the range of points, the cylinder diameter $d_{\text {real}}$ cannot generally be estimated by a local optimisation algorithm within one run, i.e., without random restarts. This only holds for cases above the indicated line. The data seems to follow well a power law

2 $$\begin{aligned} \kappa _{\text {crit}}(d_{\text {real}}) = a\,d_{\text {real}}^{-b}\,, \end{aligned}$$

where *b* is a characteristic “critical” exponent of this phase boundary. This behaviour is similar to *finite-size scaling*, a method to determine phase transitions in physical systems. Here the diameter $d_{\text {real}}$ of the cylinder plays the role of the system size. The corresponding values of *s* and *b* obtained from a fit are given in Table 1. For the right side, the data points are met by the fitting function especially well, which is mirrored by the precision of the fitting parameters. Nevertheless, both values are close to one, which appears reasonable since in the most simple ray-of-light picture, the path length of light through the cylinder should scale linearly with the diameter, hence the total absorption. Nevertheless, due to the wave nature of light and the complex geometry, the actual scaling behaviour is slightly different. Note that the value of *b* is smaller for the case of the minima appearing on the right of the global minimum, hence, this behaviour will dominate for very large values of $d_{\text {real}}$. Thus, we use this value for further analysis.

**Table 1:** Parameter values *a* and *b* of the power law given in Eq. (2) for the both sides of the energy landscape.

Side	*a*	*b*
Left	$6(2)\times 10^{-7}$	0.95(5)
Right	$1.24(2)\times 10^{-6}$	0.85(1)

Note, as we only considered one value of ${\text {Re}}(n_s)$ only, it is yet unknown whether this property also depends on the real part of the refractive index, which, as we already know, affects the periodic spacing of the local minima and hence the number of minima found per unit in parameter space.

Next, it is interesting to ask, whether the scaling behaviour Eq. (2) does not only describe the behaviour of the point where the number of minima reduces to one, but maybe the behaviour of the full curves $N_{\min }(d_{\text {real}})$. For this purpose, we consider the scaling assumption

3 $$\begin{aligned} N_{\min }(d_{\text {real}}|\kappa )= f(\kappa \, d_{\text {real}}^b), \end{aligned}$$

where *f* is an unknown “scaling” function of one argument. Since finite-size scaling holds in the limit of large sizes, we used the data for the total number $N_{\min }$ of local minima for the larger sizes $d_{\text {real}}\ge 400\, \upmu \hbox {m}$. Here the scaling according to Eq. (2) is dominated by the local minima in the right part, i.e., the power law with $b=0.85(2)$. We applied the autoScale.py package^38^ to find an independent value of *b* which leads to an optimum *data collapse* according to Eq. (3). The best agreement was found for $b = 0.857(3)$, which is compatible with the value of *b* shown in Table 1. The resulting rescaled plot is shown in the right of Fig. 1. The collapse is rather good, given the fact that the unscaled data (see inset) are very far apart. Thus, the exponent $b\approx 0.85$ seems to describe the behaviour of the full data well.

We have performed the same analysis for the case of noisy measurement data. The resulting $N_{\min }(\kappa )$ curves look very similar as for the noiseless case and also the value $b\approx 0.85$ leads to a corresponding data collapse (thus not shown here). Hence, the presence of (weak) noise alters the phase boundary and the scaling behaviour only in a negligible way.

### Algorithmic solvability

**Figure 4:**
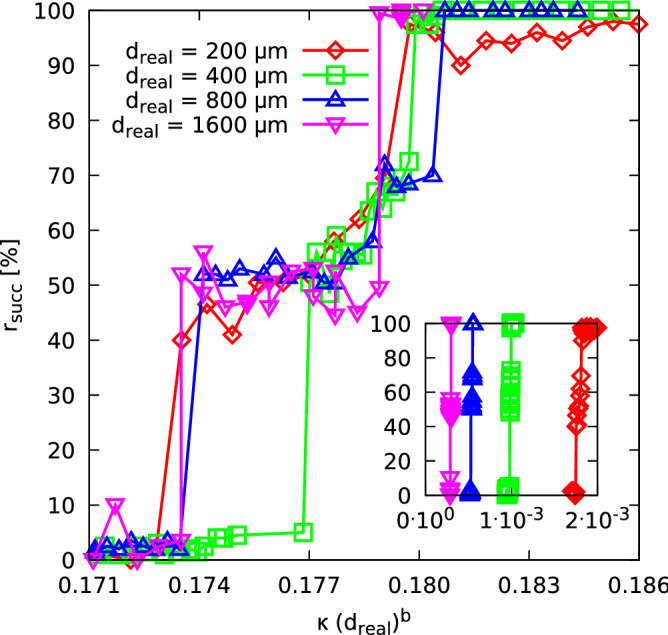
The algorithmic solvability of the optimisation problem constituted by estimating the cylinder diameter $d \equiv d_{\text {real}}$ as expressed by the fraction of correct estimations $|d_{\text {final}} - d_{\text {real}}| < 0.1\, \upmu \hbox {m}$ of all samples considered by the IRGN algorithm, with the initial values $d_0$ randomly drawn from a range of $[d_{\text {real}} - 50\, \upmu \hbox {m},d_{\text {real}} - 50\, \upmu \hbox {m}]$ (each data point is the average of 200 random initial values). As local minima vanish under increase of $\kappa$, the algorithm becomes more and more likely to terminate at the correct diameter. A rescaling with respect to the actual diameter $d_{\text {real}}$ of the ordinate axis brings the different curves for into reasonable agreement. The inset shows the different cases without rescaling. The dashed lines are to guide the eyes only.

As mentioned in the introduction of this section, the question is how the transition between transparent and less transparent behaviour affects the solvability of the optimisation problem through the IRGN algorithm. We expect, the higher number of the minima, the less probable the IRGN algorithm finds a minimum in a run with a random starting point. Hence, if $N_{\min }=1$, the algorithm should always find the solution. To investigate this issue, we considered the same diameters $d_{\text {real}}$ and absorption coefficients $\kappa$ as in the previous explicit sampling of the energy landscape.

The success rate $r_{\text {succ}}$ is shown as a function of $\kappa$ in the inset of Fig. 4. On this scale, one cannot see the shapes of the curves well. Hence, we again performed a rescaling according to $\kappa \, d_{\text {real}}^b$ on the *x*-axis, as shown in the main figure, where the value of $b = 0.86(7)$ led to the best collapse. Again, a rather nice universal behaviour is visible. Interestingly, the curves match well in the “intermediate” regime marked by a plateau of about 50% successful restarts. Note that this regime technically veils the difference between the left and right sides of the energy landscape. Anyway, we did not treat the two “sides” of the landscape here separately, since for large cylinder diameters the total number of minima is governed by those lying right to the global optimum anyway.

Please note that the results shown Fig. 4 are in detail specific for the IRGN algorithm we have used. For practical applications there may be variants which are better suited for the inverse problem studied here. Nevertheless, since the problem exhibits hard phases with many local minima, i.e., the optimisation problem is not convex, the algorithmic solvability will look in principle very similar for other algorithms, at least for the so-far known ones.

**Figure 5:**
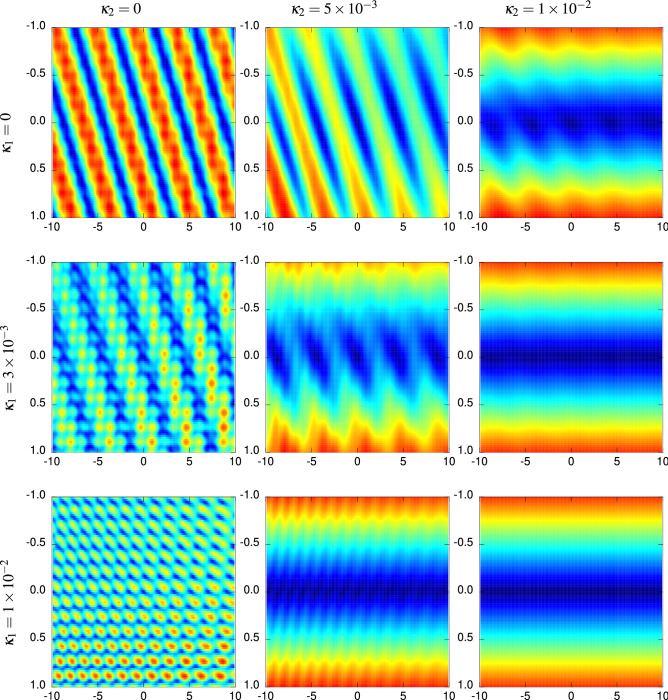
Energy landscapes for a stratified cylinder with $\vec {d}_{\text {real}} = (50,100)\, \upmu \hbox {m}$, ${\text {Re}}(n_2) = 1.457$, ${\text {Re}}(n_1) = 1.1\times {\text {Re}}(n_2)$ and various absorption coefficients $\kappa _1$ and $\kappa _2$. The colour map indicates low values of the objective function $R(\vec {d})$ in blue and high values in red. The axes show the differences $d_1-d_{1,\text {real}}$ and $d_2-d_{2,\text {real}}$ in $\upmu \hbox {m}$ units.

## Results for the two-layer fibre

For the one-layer case, the periodic structure of the energy landscapes can be rather well understood ^9^. Nevertheless, for cylinders consisting of more layers, as we will see now, the interactions between the layers lead to energy landscapes, which are not mere products of two one-layer landscapes. For this reason, we also do not expect that analytical results are straightforward to obtain, which makes the now presented numerical study even more useful.

For cylinders consisting of two layers ($r=2$, note that for real step-index fibres, the outer layer is referred to as the *cladding* while the inner layer is called the *core*), the energy landscapes are calculated over a two-dimensional support consisting of the respective layer diameters $d_1$ and $d_2$. Some sample landscapes for selected combinations of the imaginary parts $(\kappa _1,\kappa _2)=({\text {Im}}(n_1),{\text {Im}}(n_2))$ of the refractive indices $(n_1,n_2)$ of the two layers are shown in Fig. 5. For transparent (or semi-transparent) layers, the aforementioned periodicity of local extrema determines a topography dominated by parallel trench-like structures along which local minima are located, while in turn the local maxima form ridges between these trenches. It appears that this topography basically results from a kind of superposition of the respective energy landscape formed over each axis.

When altering $\kappa _2$ of the outer ($s=2$) layer, a similar transition as for the homogeneous case can be observed. This affects the presence and location of local minima along the corresponding $d_2$-axis, but also the energy landscape for the optimisation problem as a whole (as an effect of the aforementioned superposition): The vanishing of local extrema along the $d_2$-axis gradually breaks down the trench topography towards a relief where only one large trench along the $d_1$-axis is present. In the intermediate regime, the aforementioned “local” trenches are present on the slopes leading towards the “global” trench. This is demonstrated by the first row of images in Fig. 5. In turn, for an increase of the imaginary part ${\text {Im}}(n_1) = \kappa _1$ of the inner layer ($s=1$), the norm behaves differently as function of $(d_1,d_2)$. As can be seen from the first column of Fig. 5, the trench structure appears to break down in favour of a kind of intersecting parallel lines. When, however, $\kappa _2$ is increased as well, it turns out that the overall topology of $R(\cdot )$ becomes similar to the single-trench case that can also be observed for a sufficiently high value of $\kappa _2$ alone. This is possibly related to the fact that the scattering intensities are lowered even further by absorption in both layers already.

Regarding the question how these observations affect the solvability of the optimisation problem consisting in finding $\vec {d}_{\text {real}} \equiv (d_{1,\text {real}},d_{2,\text {real}})$ from $R(\vec {d})$, the following can be stated from the observations: When altering ${\text {Im}}(n_2)$ sufficiently to produce the “global” trench only, local minima are only present on the bottom of this trench and hence located at $d_2 \equiv d_{2,\text {real}}$ along the $d_1$-axis. In this notion, the 2-dimensional problem thus breaks down to a 1-dimensional one, which obviously can be solved far more easily. Note also that any local optimisation algorithm such as—once again—IRGN always leads into this global trench, so the diameter of the outer layer is easily determined. Judging from this, the dependence of the complexity of the optimisation problem on $\kappa _2$ is probably more intuitive, so we present below the case with $\kappa _1 = 0$ first.

**Figure 6:**
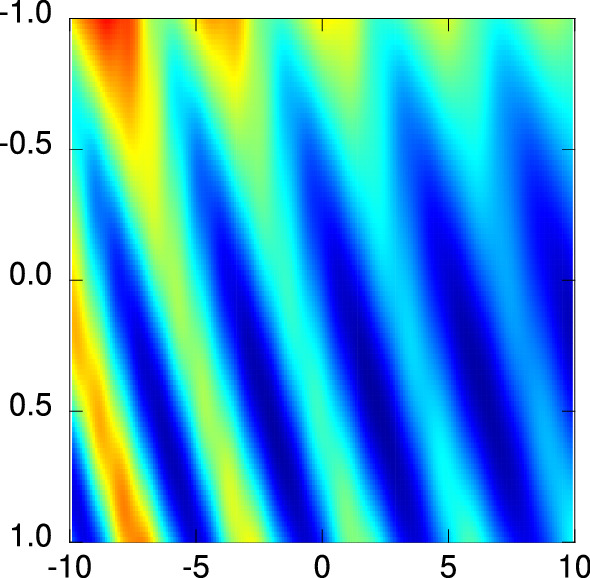
Results regarding the two-layered cylinder with diameters for the case $\kappa _1=3\times 10^{-3}$ and $\kappa _2=5\times 10^{-3}$ in the presence of noise. The axes show the differences $d_1-d_{1,\text {real}}$ and $d_2-d_{2,\text {real}}$ in $\upmu \hbox {m}$ units.

We also studied the case with 1% noise in the measurement data. For most cases, the energy landscapes changed only slightly. Nevertheless, sometimes we observed a notable change, as for the case $\kappa _1=3\times 10^{-3}$ and $\kappa _2=5\times 10^{-3}$, which is shown in Fig. 6. The structure of the energy landscape changes slightly in comparison to the corresponding case shown in Fig. 6 (plot in the centre). In particular, the position of the global minimum is not any longer located at the true radii, in particular for the inner radius $d_1$. This is similar to the one-layer noisy case investigated above. Nevertheless, as in the one-layer case, the overall parameter-controlled behaviour of the energy landscapes does not change, thus, we proceed by focusing on the zero-noise case.

**Figure 7:**
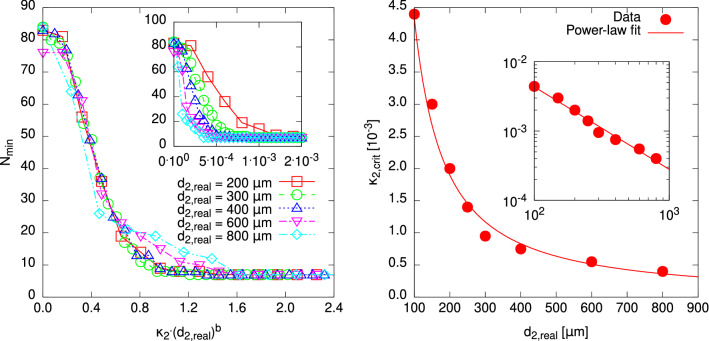
Results regarding the two-layered cylinder with diameters $d_{s,\text {real}} = (50\, \upmu \hbox {m}, d_{2,\text {real}})$ under variation of the imaginary part ${\text {Im}}(n_2) = \kappa _2$ of the refractive index $n_2$ of the outer layer. (left) The numbers $N_{\min }$ of found local minima of the energy landscape formed by $R(\vec {d})$. For various outer layer diameters $d_{2,\text {real}}$, the curves could be brought into agreement by scaling the ordinate axis according to Eq. (3) with the scaling parameter $b = 1.26(2)$. (left, inset) The unscaled data, the lines are to guide the eyes only. (right) The value $\kappa _{2,\text {crit}}$ beyond which the number of found local minima attained the minimum value, which is here seven due to the value of the diameter of the inner layer, plotted as a function of the outer layer diameter $d_{\text {2,real}}$. Once again, a power-law dependence according to Eq. (2) could be established. (right, inset) The data on a double-logarithmic scale.

For the actual measurements, we considered a stratified cylinder with a core diameter $d_{1,\text {real}} = 50\, \upmu \hbox {m}$ and real refractive indices ${\text {Re}}(n_s) = (1.1\times 1.457, 1.457)$, i.e., with a real refractive index difference of 10% between inner and outer layer. The imaginary part of the refractive index of the core was ${\text {Im}}(n_1) = 0$. The imaginary part ${\text {Im}}(n_2)$ of the refractive index of the outer layer or cladding was varied as the control parameter. For different outer diameters $d_{2,\text {real}}$, we sampled and counted the local minima of $R(\vec {d})$. The whole considered region of diameters was $\vec {d} = \vec {d}_{s,\text {real}} \pm (0.5, 5)\, \upmu \hbox {m}$ of the actual layer diameters, i.e., a rectangle of size $(1 \times 10)\, \upmu \hbox {m}^2$. We found it sufficient to sample an actual grid within this region only for the initial value ${\text {Im}}(n_2) = 0$ for each cladding diameter $d_{2,\text {real}}$. When applying the IRGN algorithm to the intensity pattern for $(d_{s,\text {real}},n_s)$ at each point of the grid, the algorithm seeks out the local minima, and for the next imaginary value ${\tilde{{\text {Im}}}}(n_2) > 0$, those local minima can be used as the starting points, and so on recursively. This proved to be helpful in terms of computation time, since the whole grid did not need to be considered for the further increase of $\kappa _2$.

From that on, the evaluation of data is straightforward and analogous to the homogeneous case. Once again, we evaluate the behaviour of the number of local minima as a function of $\kappa _2$ and try to collapse the curves with respect to the actual outer diameter $d_{2,\text {real}}$ according to the scaling relation of Eq. (3), yielding a scaling parameter *b*. The result is shown in the left plot of Fig. 7, with the best found parameter value $b = 1.26(2)$ differing considerably from that of the homogeneous case. It appears physically meaningful to attribute this difference to the dimension of the optimisation problem, with this representing a stratified cylinder of $r=2$ layers instead of $r=1$ in the homogeneous case.

The usage of the IRGN algorithm in order to locate the minima that are reachable from the considered $\vec {d}$ region also allows us to to make statements about the “global” solvability of the two-dimensional optimisation problem with this “local” algorithm. As may be guessed from the final numbers of found local minima in Fig. 7 (left) being 7 for all considered cases, it probably is of no surprise that the success ratio of the IRGN algorithm is 14.28% or $\frac{1}{7}$, which obviously is rather bad, but much higher than the values obtained at ${\text {Im}}(n_2) = 0$, which range from ca. 0.87 to 1.30%. This once more highlights the way that the transition from transparency to weak transparency affects the difficulty of algorithmic diameter estimation.

However, we will not evaluate the success ratio for this case quantitatively any further. Instead, we once more seek to establish a relation regarding a “critical” point $\kappa _{2,\text {crit}}$ concerning the second layer, which marks the onset of the region where the minimum number of local minima is observed. This is analogous to the the property evaluated for the homogeneous case in Fig. 3, so we use the power-law of Eq. (2) again. The resulting plot is shown in the right of Fig. 7. The found exponent in the power-law fit is $b = 1.19(6)$, which does not exactly meet that obtained from the scaling of the numbers of local minima, but both values are of a similar magnitude, and their errors overlap. A better agreement might be found if $\kappa _2$ was sampled with higher precision near $\kappa _{2,\text {crit}}$. But anyway we find this to be a corroboration of the scaling exponent being in the range of $b = 1.2$ for cylinders consisting of two layers.

**Figure 8:**
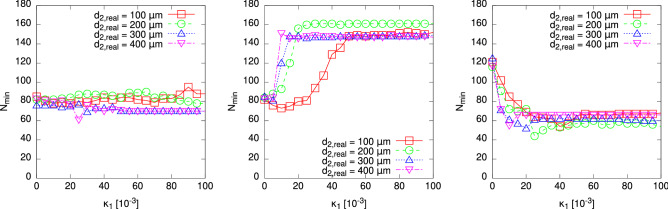
Dependence of the number $N_{\min }$ of local minima of the objective function $R(\cdot )$ found within an interval of $(d_1,d_2) \in [10,1]\, \upmu \hbox {m} \times [10,1]\, \upmu \hbox {m}$ on the absorption coefficient $\kappa _1 \equiv {\text {Im}}(n_1)$ of the inner layer ($s=1$) of a two-layered cylinder. For each ratio $\frac{d_1}{d_2}$ of layer diameters (left: $\frac{d_1}{d_2} = 0.2$, centre: $\frac{d_1}{d_2} = 0.6$, right: $\frac{d_1}{d_2} = 0.9$), a number of different values of $\kappa _1$ was considered. It turns out that the layer diameter ratio generally determines the behaviour of $N_{\min }$, as can be seen from the fact that $N_{\min }$ attains similar values for different outer layer diameters $d_2$. Lines are guides to the eyes only.

Regarding the alteration of the inner layer absorption coefficient $\kappa _1 = {\text {Im}}(n_1)$, it has been stated before that the optimisation problem constituted in finding the global minimum of $R(\cdot ) = ||F(\vec {x}|\vec {d}^{(n)}) - u_\infty ||$ does not become easier when altering $\kappa _1$ alone. Instead, due to the changes of the topography of the energy landscape (as shown in Fig. 5), the number of local minima seems to grow. This however might be an effect which depends on other properties of the scattering cylinder as well, namely, the ratio $\frac{d_1}{d_2}$ of the respective layer diameters. In order to investigate this, the number $N_{\min }$ of local minima was obtained by sampling within a $[10,1]\, \upmu \hbox {m} \times [10,1]\, \upmu \hbox {m}$ rectangle of the $(d_1,d_2)$ support, again by usage of the IRGN algorithm in order to discriminate all local minima that are reachable with initial values within this area. For the same ratio $\frac{d_1}{d_2}$, a number of outer diameters $d_2$ were considered, and with that $N_{\min }$ was measured for a certain range of $\kappa _1$. Figure 8 thus demonstrates two peculiarities related to these parameters: First, it appears that the number of minima $N_{\min }$ is generally ruled by the diameter ratio $\frac{d_1}{d_2}$, as the numbers for different outer layer diameters $d_2$ roughly agree for sufficiently large values of $\kappa _1$ or at least vary within a common range. This is especially striking for higher values of $\frac{d_1}{d_2}$. Second, the behaviour of $N_{\min }$ itself as a function of $\kappa _1$ is interesting. For small ratios such as $\frac{d_1}{d_2} = 0.2$ (left plot of Fig. 8), $N_{\min }$ varies within a certain range, while for larger values such as $\frac{d_1}{d_2} = 0.6$ (centre plot of Fig. 8) a rapid increase towards a constant value can be observed, which by the way appears to happen earlier for smaller values of $d_2$. With a large ratio such as $\frac{d_1}{d_2} = 0.9$ (right plot of Fig. 8), however, the number of minima drops with increasing $\kappa _1$. A possible dependence of the steepness of this drop on $d_2$ is also visible. Yet the “final” constant values are not nearly as low as those for increasing $\kappa _2$ only, which hints again on the more complex energy landscapes as demonstrated in the corresponding images shown in Fig. 5.

**Figure 9:**
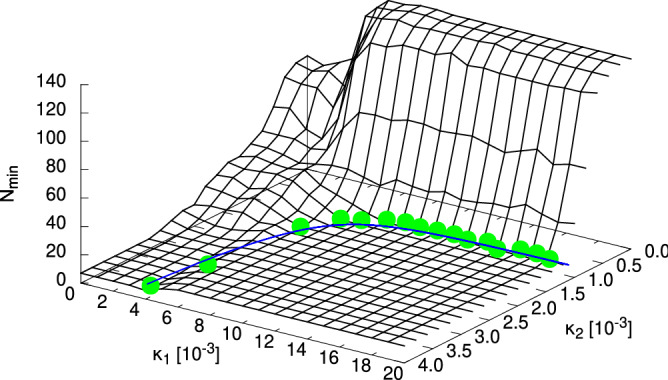
Phase diagram of the optimisation problem for the $r=2$ layer cylinder with layer diameters $\vec {d} = (50,125)\, \upmu \hbox {m}$. The points and the curve in the $(\kappa _1,\kappa _2)$ plane mark the border between the harder (high number $N_{\min }$ of local minima) and the simpler (low $N_{\min }$) phase of the optimisation problem. On the simple side, the diameter $d_2$ of the outer layer is easy to determine, and only $d_1$ requires more numerical effort. On the harder side, both diameters are difficult to estimate. A power-law dependence of the phase-boundary curve $\kappa _2(\kappa _1)$ could be found, depicted by the green (light) dots and the (blue) curve. Please note that the blue dots represent the lowest values of $N_{\min }$ found under variation of $\kappa _2$ at a given $\kappa _1$. Note also that particularly low values of $\kappa _1 \le 4\times 10^{-3}$ appear to be behave slightly non-monotonously, under further decrease of $\kappa _1$. Probably, here the sampling resolution was still not high enough to resolve all details. Since we are not interested in small details of the phase diagram, the sampling resolution is sufficient for our purpose here.

Finally, we move over to the concurrent alteration of the absorption coefficients $\kappa _1$ and $\kappa _2$ of both diameters. Again, the observable quantity is simply the number $N_{\min }$ of local minima found within the given range for $\vec {d} = (50,125)\, \upmu \hbox {m}$, $n_2 = 1.457$ and $n_1 = 1.1\times n_2$. The evaluation for this is straightforward, as through increasing $\kappa _2$ the energy landscape becomes less complex even for $\kappa _1 > 0$. Therefore, it is sufficient to start at one value of $\kappa _1$, figure out the local minima for this case by means of the IRGN algorithm from the full grid of starting points and then increase $\kappa _2$ afterwards, starting at the previously found minima each time. Then, for each value of $\kappa _1$ the value of $\kappa _2$ can be determined at which $N_{\min }$ has reached a constant value, i.e., $N_{\min }$ remains at this value for higher values of $\kappa _2$. With these values, a curve of pairs $(\kappa _1,\kappa _2)$ can be constructed which so to say represents the “phase diagram” for this case, as shown in Fig. 9. One side of it represents the “hard” phase in which further local minima are present, while the other side (and the line itself) marks the much “easier” phase in which all local minima are situated along the aforementioned main trench along the $d_1$ axis, i.e., $d_2$ is easy to determine and only $d_1$ needs additional effort. A detailed inspection of the phase diagram exhibits that the easier phase is characterised by about $N_{\min } \approx 7...12$ local minima. Analysing the corresponding values reveals that the shape of the phase boundary described as dependence of $\kappa _2$ on $\kappa _1$ can actually be expressed in terms of another power-law dependence, i.e., $\kappa _2(\kappa _1) = c + \alpha \cdot \kappa _1^{-\gamma }$. The resulting parameters of the function curve shown in Fig. 9 are $c = 109(5) \times 10^{-5}$, $\alpha = 11(8) \times 10^{-11}$ and $\gamma = 3.5(1)$. It is likely the case that these values actually depend on those layer diameters $\vec {d}$ of the cylinder, which have been fixed in this case, since phase boundaries are usually non-universal quantities, see e.g. the dependence of $\kappa _{\text {crit}}$ on $d_{\text {real}}$ in the homogeneous case.

## Conclusion and outlook

In this work we have studied the determination of diameters of *r* layers for (semi-)transparent cylinders, e.g., optical waveguides, from lateral diffraction patterns. This is an inverse problem which can be stated as an optimisation problem for a norm *R* measuring the difference between a pattern resulting from a numerical simulation and the actually measured pattern. In the present work, the latter one is also obtained from a simulation for given (known) diameters. In particular we have investigated the *energy landscapes*, which describe the dependence of *R* on the values for the diameters, for given values of the complex refractive indices $n_s$. We find that the nature of these landscape changes, depending on the values of $n_s$, between a region where the landscape is dominated by one global minimum (or few minima), and a region where many local minima appear (where the number grows with the system size). The existence of many local minima makes it much harder for a Gauss-Newton algorithm with random restarts to actually find the global optimum. Thus, already in a very broad sense, one can classify the two regions as *easy* and *hard* phases. Note that in the hard region, when varying any of the *r* diameter variables, one will encounter multiple local minima, as we have shown explicitly for the case $r=2$. Thus, when identifying the number of variables to optimise as “size” of the system, as it is done in classical optimisation problems like *Travelling Salesperson* and *Satisfiability*, having roughly ${\overline{N}}_{\min }$ local minima in each diameter “direction” or “dimension”, will lead to $({\overline{N}}_{\min })^r$ local minima for a system with *r* layers. Thus, the easy-hard transition observed for the present problems corresponds exactly to a change from typical polynomial to typical exponential running time as a function of *r* as it has been observed in the classical cases. Thus, with the present work, we have shown that such easy-hard transitions do not only occur for abstract models, but can be observed also for real optimisation problems which are of technological relevance. Investigating these transitions might help in understanding the actual technological problems better or guide the selection of suitable optimisation algorithms.

We were able to quantify this transition for homogeneous ($r=1$ layer only) and two-layered ($r=2$) cylinders. The actual “critical” value of the imaginary part $\kappa$ depends on the diameters of the layers, but seems to be described well by a power law defined by an exponent *b*. It is characteristic for the dimension of the optimisation problem, as we found differing values of $b \approx 0.86$ for the homogeneous case and $b \approx 1.2$ for the outer layer $d_2$ of a two-layered cylinder. This exponent not only determines the actual transition towards easy solvability but also governs the full behaviour of the numbers of local minima upon the raising $\kappa$. The description of the easy-hard transition by an exponent resembles the characterisation of physical phase transition by critical exponents, as it has also been used for the analysis of easy-hard transition for abstract model systems.

We also studied for some parameter combinations, for the one-layer as well as for the two-layer case, the influence of the presence of noise. We observed that in some cases the estimations from even the best algorithms may become somehow off, because the locations of the global minima change. Also changes in details of the shape of the energy landscape are visible. Nevertheless, the overall picture of the easy-hard transitions of the diameter-determination problem is not altered by the presence of weak noise. Clearly, for (too) strong noise, the determination of the parameters will be prohibited at all, unless one averages over many measurements to make the noise effectively weak again. Thus, this case also not relevant for the present study.

Several points have been deliberately left out in this work which may be starting points for further research. Mainly, we kept the real parts ${\text {Re}}(n_s)$ of the refractive indices fixed, although we do not expect the transition to depend on this as much, as earlier work showed that the relative refractive index $m_s = \frac{m_s}{m_{s+1}}$ affects the spacing of the local minima, but not the general characteristic of the energy landscape^9^. Regarding two-layered cylinders, we focused on the outer layer $s = r \equiv 2$ only. It might seem interesting to to investigate the core layer, however our notion is that possibly no transition exists for this layer—as mentioned, preliminary research did not find any cases where ${\text {Im}}(n_1)$ resulted in a global minimum only for this layer. On the contrary, these preliminary research showed that increasing $\kappa _1$ does not eliminate local minima, but rather changes the energy landscape in such a way that the number of minima actually rises. This however may be different for ${\text {Re}}(n_1) \approx {\text {Re}}(n_2)$ and $\kappa _1 > \kappa _2$, i.e., a cylinder that becomes more absorbing towards its centre.

Another open question is the behaviour for more layers, i.e., $r > 2$. With the previous statements in mind, the numbers of local minima affected by alteration of the refractive index ${\text {Im}}(n_r)$ of the outermost layer appear to be the most relevant quantities when it comes to the dimensional reduction of the optimisation problem. In such cylinders, however, layers exist that are neither the innermost nor the outermost. We cannot yet answer whether these “intermediate” layers behave like the outermost layer and expose a transition between hard and easy that may be utilised for the simplification of the optimisation problem, or whether they are more like the core layer. Also, it could be interesting to investigate whether the local minima exhibit a kind of clustering in the energy landscape, as it has been observed numerically in the case of classical optimisation problems^39,40^, and where it corresponds to the so-called *replica symmetry breaking*^27,41,42^. Nevertheless, for the present problem it would be much harder to study, because every single evaluation of the target function *R*, which requires the full solution using the Lorenz–Mie theory, is much harder than the evaluation of the energy function for the classical combinatorial optimisation problems.

The determination of radii of optical wave guides from measured inference patters is only one example of an inverse problem. Many other inverse problems exist like computer tomography, analysis of elementary particle collisions, or analysis of stellar objects. Thus, we expect that for other types of inverse problems, different problem ensembles and applied optimisation algorithms many types of easy-hard phase transitions and various organisations of energy landscapes will occur. This expectation is driven by the results obtained in the classical field of phase transitions in combinatorial optimisation problems, where quite different structures of energy landscapes and corresponding phase transitions are observed for problems as different as *Travelling Salesperson*, *Vertex Cover* or *Satisfiability*.

Finally, our study represents just one example, where real-world technological optimisation problems exhibit easy-hard transitions, beyond so-far studied classical problems. Thus, it could be of interest to identify other realms of science and engineering where such optimisation problems exhibit easy-hard transitions and to investigate whether they tell us something about the nature of the problems and help one to choose the best-suited algorithms to solve them.

## Supplementary information

Supplementary material 1
