# Supplementary material for: Easy-hard phase transition in parameter estimation for optical waveguides
Source: Sci Rep. 2020 Oct 15;10:17336. doi: 10.1038/s41598-020-74366-5 (PMC7562953; doi:10.1038/s41598-020-74366-5)
Supplement: Supplementary file 1 — Supplementary material 1 [file 41598_2020_74366_MOESM1_ESM.pdf]

Easy-hard phase transition in parameter estimation for optical waveguides: supplementary information

Gunnar Claussen[1,*] **and Alexander K. Hartmann**[1,+]

[1]Institut für Physik, Carl von Ossietzky Universität Oldenburg, D-26129 Oldenburg, Germany
[*]gunnar.claussen1@uni-oldenburg.de
[+]these authors contributed equally to this work

ABSTRACT

These are the supplementary informations to the paper "Easy-hard phase transition in parameter estimation for optical waveguides". This document is intended to specify methodic details omitted in the original publication regarding the formalism used to numerically calculate the scattering intensities $F(\vec{x}|q)$ on a infinite circular cylinder and the iterative regularized Gauss-Newton (IRGN) algorithm that allows for detection of local minima in the objective function $||F(\vec{x}|q) - F(\vec{x}|q_{\text{real}})||_2$ of the optimization problem given by seeking the correct values q_{real} of the parameters q. As these methods are largely identical to those used in the previous publication on diameter estimation[1], we leave them here as supplementary information instead of including them in the actual publication.

Calculation of scattering patterns

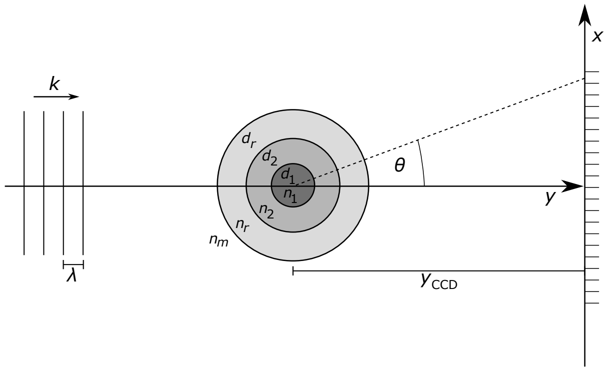

Figure 1. General setup for the generation of scattering patterns. Monochromatic light of wavelength λ illuminates the scattering cylinder (r layers of diameter d_s and refractive index n_s, respectively) perpendicularly. The scattered light is then detected at a number of coordinates $\vec{x}$ representing CCD cells arranged in a line.

Consider the scattering setup shown in Fig. 1. The theoretical background allowing for numerical calculation of the scattering pattern $I(\vec{x})$ is founded on Lorenz-Mie theory,[2] as described by textbooks on optics.[3,4] In general, this theory presents solutions of the Maxwell equations in presence of a cylindrical object, whose dielectric properties expressed through the wave number k require certain boundary conditions to be fulfilled. This is done by transformation into cylindrical coordinates (ρ, φ, z) and expansion into Bessel functions. Due to the assumptions of given geometry, i.e. perpendicular incidence of light and orientation of scattering object and detection line in a plane, only the parallel polarized component $E_{s\|}$ of the scattered electrical field $\vec{E}_s$ is of interest, which is given by the expansion

$$E_s(\vec{x}_p) \equiv E_{s\|}(\vec{x}_p) = -\sum_{-\infty}^{\infty} E_n b_n N_n(\vec{x}_p), \tag{1}$$

with $E_n = E_0 \frac{(-i)^n}{k}$ with respect to a basic amplitude E_0. N_n are the so-called vector-harmonic generating function $N_n(\vec{x}_p)$ defined by

$$N_n(\vec{x}_p) = \sqrt{k} \cdot k \cdot Z_n(\rho) \cdot e^{in\varphi}. \tag{2}$$

In this, $Z_n \equiv J_n(\cdot)$ denotes the Bessel function, and the argument $\rho = r\sqrt{k^2 - h^2}$ simplifies to $\rho = r \cdot k$, since the expression $h = -k\cos\zeta = 0$ holds for $\zeta = 90°$. Information about the scattering cylinder only enters into Eq. (1) through the definition of the scattering coefficients b_n. To shorten explanations here, we use a general recursive definition that allows for homogenous cylinders as well as for stratified cylinders.[4,5] This definition is given for the outermost ($s = r$) layer according to:

$$b_n = \frac{m_r J_n(x_r)[J_n'(m_r x_r) + T_n^{r-1} N_n'(m_r x_r)] - J'n(x_r)[J_n(m_r x_r) + T_n^{r-1} N_n(m_r x_r)]}{m_r H_n(x_r)[J'n(m_r x_r) + T_n^{r-1} N_n'(m_r x_r)] - H_n'(x_r)[J_n(m_r x_r) + T_n^{r-1} N_n(m_r x_r)]} \tag{3}$$

In this $J_n(\cdot)$, $H_n(\cdot)$ and $N_n(\cdot)$ denote the Bessel, Hankel and Neumann functions, respectively. x_s is the size parameter $x_s = k_{s+1} \cdot \frac{d_s}{2}$ for the s-th layer and $m_s = \frac{n_s}{n_{s+1}}$ is the corresponding relative refractive index. Now, for stratified cylinders the auxiliary quantities T_n^{r-1} come into play. Starting at $r-1$, this quantity is recursively calculated by

$$T_n^s = \frac{m_s J_n(x_s)[J_n'(m_s x_s) + T_n^{s-1} N_n'(m_s x_s)] - J_n'(x_s)[J_n(m_s x_s) + T_n^{s-1} N_n(m_s x_s)]}{m_s N_n(x_s)[J_n'(m_s x_s) + T^{s-1} N_n'(m_s x_s)] - N_n'(x_s)[J_n(m_s x_s) + T_n^{s-1} N_n(m_s x_s)]}. \tag{4}$$

For the innermost or $s = 1$ layer, $T_n^0 = 0$ is used and hence ends this recursion. Thus, the whole calculation of the intensity pattern $I(\vec{x})$ subsumed by the expression $F(\vec{x}|q_n)$ basically consists only of calculation of the vector-harmonic generating functions according to Eq. (2), the scattering coefficients according to Eq. (3) (occasionally also with the recursion of Eq. (4)), the subsequent summation of Eq. (1) and, finally, the calculation of the absolute by

$$I(\vec{x}) = \mathrm{Re}\left(E_{\mathrm{total}}(\vec{x})\right)^2 + \mathrm{Im}\left(E_{\mathrm{total}}(\vec{x})\right)^2. \tag{5}$$

Note that the sum in Eq. (1) has the index $n \in [-\infty, \infty]$. Technically, summation up to a truncation number $n_{\max} = \lceil x_r + 4 \cdot \sqrt[3]{x_r} + 2 \rceil$ is sufficient.[3]

Gauss-Newton algorithm

Usage of the iteratively regularized Gauss-Newton (IRGN) is in particular useful for cases where the Euclidean norm of a vector has to be minimized, because one can work directly with the vector entries, see below (here the vectors are indxed by the measured positions $\vec{x}$ and contain N entries). In the outline of the algorithm stick to the description given by Hohage and Schormann.[6] We would like to stress the role of the operator F, which, with respect to the scattering formalism outlined above, might be a bit more illustrative. In the original publication, the task is to find the optimum parameter set q for a possibly noisy input pattern u_∞^δ. To do so, the expression

$$||F'(\vec{x}|q_n)h_n + F(\vec{x}|q_n) - u_\infty^\delta||_2 + \alpha_n ||h_n + q_n - q_0||_2 \stackrel{!}{=} \min \tag{6}$$

has to be minimized, with the index n denoting the current iteration step. $F'(\vec{x}|r_n)$ is the Fréchet derivative of F for the current parameter value q_n. For the present work, the input pattern will also be obtained numerically for a given reference set q_{real} of parameters, i.e., $u_\infty^\delta \to F(\vec{x}|q_{\mathrm{real}})$, as above. Now, h_n is the step alteration of the current iteration and q_0 is the initial value of the parameter set. The coefficient α_n is called the *regularization factor*. Technically, this applies a weight to the expression: For large α_n the second summand of Eq. (6) containing the distance between the current and initial value of the parameters q_n is prioritized. Usually, α_n is decreased over the course of the algorithm according to $\alpha_n = \alpha_0 \cdot r^{-n}$ with $r < 1$. Hence, the longer the algorithm lasts, the more similar it gets to the usual Gauss-Newton algorithm.

The IRGN algorithm can be applied by reforming (6) into an explicit expression for the step alteration h_n given by

$$h_n = -(F'(\vec{x}|q_n)^* F'(\vec{x}|q_n) + \alpha_n I))^{-1} (F'(\vec{x}|q_n)^* (F(\vec{x}|q_n) - u_\infty^\delta) + \alpha_n (q_n - q_0)), \tag{7}$$

where $\cdot^*$ denotes the conjugate transpose matrix, $\cdot^{-1}$ the inverse matrix, and I is the identity matrix. For clarity, please note the dimensionalities within this equation: $F(\vec{x}|q_n)$ and u_∞^δ are vectors of length X (corresponding to the number of observation

points $\vec{x}$), while q_n and q_0 are tuples with the parameters to be optimized, i.e. have a size equal to the number of cylinder layers r. Thus, the derivatives $F'(\vec{x}|q_n)$ have the dimension $X \times r$, and the multplication $F'(\vec{x}|q_n)^* F'(\vec{x}|q_n)$ then has the dimension $r \times r$. Conversely the dimension of $F'(\vec{x}|q_n)^*(F(\vec{x}|q_n) - u_\infty^\delta)$ and the whole second parenthesis is $r \times 1$. This ultimatey results in the whole expression being of a dimension corresponding to the number of variables, i.e. r. Thus, one iteration of the IRGN algorithm incorporates the following steps only:

1. Calculate $F(\vec{x}|q_n)$ and $F'(\vec{x}|q_n)$.
2. Calculate h_n according to Eq. (7).
3. Update q_n by $q_{n+1} = q_n + h_n$.
4. Update α_n.

The original termination condition for the algorithm is the norm $||F(\vec{x}|q_n) - u_\infty||_2$ dropping below a given threshold, thus hinting that the IRGN algorithm implicitly seeks out minima. As we cannot expect this condition to be fulfilled for local minima, other conditions seem feasible, such as allowing for a maximum number of iterations only or a threshold for the step length $||h_n||_2$. Also, the algorithm requires explicit calculations of $F(\vec{x}|q_n)$ and $F'(\vec{x}|q_n)$, which – with $F(\cdot)$ being the formalism outlined by Lorenz-Mie theory – is numerically intricate and dominates the actual computational effort. Finally, it should be remarked that analytical equations for $F'(\vec{x}|q_n)$ have been calculated for homogenous cylinders only, while for stratified cylinders we rely on numerical differentiation instead.[1]

References

1. Claussen, G. & Blohm, W. Determination of optical fiber layer parameters by inverse evaluation of lateral scattering patterns. In *Optical Measurement Systems for Industrial Inspection XI*, vol. 11056, 110561B (International Society for Optics and Photonics, 2019).

2. Mie, G. Beiträge zur Optik trüber Medien, speziell colloidaler Metallösungen. *Ann. Phys.* **IV**, 377–425 (1908).

3. Bohren, C. F. & Huffman, D. R. *Absorption and scattering of light by small particles* (John Wiley & Sons, New York, 1983).

4. Quinten, M. *Optical properties of nanoparticle systems: Mie and beyond* (Viley-VCH, Weinheim, 2011).

5. Sinzig, J. & Quinten, M. Scattering and absorption by spherical multilayer particles. *Appl. Phys. A* **58**, 157–162 (1994).

6. Hohage, C. & Schormann, T. A Newton-type method for a transmission problem in inverse scattering. *Inverse Probl.* **14**, 1207–1227 (1998).
